# Periodontal pathogens promote cancer aggressivity via TLR/MyD88 triggered activation of Integrin/FAK signaling that is therapeutically reversible by a probiotic bacteriocin

**DOI:** 10.1371/journal.ppat.1008881

**Published:** 2020-10-01

**Authors:** Pachiyappan Kamarajan, Islam Ateia, Jae M. Shin, J. Christopher Fenno, Charles Le, Ling Zhan, Ana Chang, Richard Darveau, Yvonne L. Kapila

**Affiliations:** 1 Department of Orofacial Sciences, School of Dentistry, University of California, San Francisco, CA, United States of America; 2 Department of Periodontics and Oral Medicine, School of Dentistry, University of Michigan, Ann Arbor, MI, United States of America; 3 Department of Oral Medicine and Periodontology, Mansoura University, Mansoura, Egypt; 4 Department of Cariology, Restorative Sciences, and Endodontics, School of Dentistry, University of Michigan, Ann Arbor, MI, United States of America; 5 Department of Epidemiology, School of Public Health, University of Michigan, Ann Arbor, MI, United States of America; 6 Biologic and Materials Sciences, School of Dentistry, University of Michigan, Ann arbor, MI, United States of America; 7 Department of Periodontics, Oral Health Sciences, School of Dentistry, University of Washington, Seattle, WA, United States of America; University of California, Davis, UNITED STATES

## Abstract

Epidemiological studies reveal significant associations between periodontitis and oral cancer. However, knowledge about the contribution of periodontal pathogens to oral cancer and potential regulatory mechanisms involved is limited. Previously, we showed that nisin, a bacteriocin and commonly used food preservative, reduced oral cancer tumorigenesis and extended the life expectancy in tumor-bearing mice. In addition, nisin has antimicrobial effects on key periodontal pathogens. Thus, the purpose of this study was to test the hypothesis that key periodontal pathogens (*Porphyromonas gingivalis*, *Treponema denticola*, and *Fusobacterium nucleatum*) promote oral cancer via specific host-bacterial interactions, and that bacteriocin/nisin therapy may modulate these responses. All three periodontal pathogens enhanced oral squamous cell carcinoma (OSCC) cell migration, invasion, tumorsphere formation, and tumorigenesis *in vivo*, without significantly affecting cell proliferation or apoptosis. In contrast, oral commensal bacteria did not affect OSCC cell migration. Pathogen-enhanced OSCC cell migration was mediated via integrin alpha V and FAK activation, since stably blocking alpha V or FAK expression abrogated these effects. Nisin inhibited these pathogen*-*mediated processes. Further, *Treponema denticola* induced TLR2 and 4 and MyD88 expression. Stable suppression of MyD88 significantly inhibited *Treponema denticola*-induced FAK activation and abrogated pathogen-induced migration. Together, these data demonstrate that periodontal pathogens contribute to a highly aggressive cancer phenotype via crosstalk between TLR/MyD88 and integrin/FAK signaling. Nisin can modulate these pathogen-mediated effects, and thus has therapeutic potential as an antimicrobial and anti-tumorigenic agent.

## Author summary

Oral squamous cell carcinoma (OSCC), a subset of HNSCC, is the most common malignant oral neoplasm. Risk factors, including smoking, alcohol consumption and human papilloma virus (HPV) infection alone have not been sufficient in explaining the incidence and aggressive behaviors of OSCC. Thus, other factors, such as oral periodontal pathogens may play an important role in OSCC tumor development, progression and metastasis, yet this has not been well explored. Here, we test whether periodontal pathogens promote oral cancer tumorigenesis, and whether bacteriocin/nisin treatment may modulate these responses. We found that periodontal pathogens promote cancer aggressivity via crosstalk between integrin/FAK and TLR/MyD88 signaling pathways that is reversible by bacteriocin/nisin treatment. Our study offers direct evidence that a bacteriocin, nisin has a broad therapeutic potential as an anti-cancer agent, and as an inhibitor of pathogen-mediated carcinogenesis.

## Introduction

Head and neck squamous cell carcinoma (HNSCC) is one of the most common cancers worldwide and it is a major health burden [1–3]. Each year, there are more than three hundred thousand new cases of HNSCC around the world [2]. Currently, the gold standard for diagnosis of HNSCC requires a surgical biopsy followed by histopathological examination [4]. Oral squamous cell carcinoma (OSCC), a subset of HNSCC, is the most common malignant oral neoplasm. OSCC accounts for 90% of all oral malignancies, and it has a poor 5-year survival rate that has not changed in decades [5, 6]. As risk factors, smoking, alcohol consumption and human papilloma virus (HPV) infection have been implicated [5–7]. However, these risk factors alone have not been sufficient in explaining the incidence and aggressive behaviors of OSCC. Thus, other factors, such as oral dysbiosis may play an important role in OSCC tumor development, progression and metastasis, yet this has not been well explored.

Periodontitis, a chronic inflammatory disease mediated by a bacterial dysbiosis and unfavorable host-bacterial interactions, is characterized by destruction of periodontal tissues. *Treponema denticola*, *Porphyromonas gingivalis* and *Tannerella forsythia* appear in late stages of oral biofilm development and comprise the bacterial “red complex” that is considered pathogenic in the etiology of periodontal disease [8]. Other periodontopathogenic bacteria have been proposed for inclusion in the red complex [9, 10]. Recently, the implementation of omics technologies, specifically high-throughput sequencing technologies, such as 16S rRNA and shotgun metagenomic sequencing have enabled a more holistic approach to the assessment of host oral microbiota. Previous studies using 16S rRNA and shotgun sequencing have confirmed significant differences in microbial community structures between health and periodontitis [11, 12]. Recent omics studies have increased our knowledge relative to periodontal pathogens at the genus level, specific to progressing periodontitis *(Bulleidia and Olsenella)* and stable periodontitis *(Campylobacter and Eubacterium)*. In addition, at the species level, *Bulleidia extructa*, *Eubacterium infirmum*, *Fusobacterium periodonticum*, *Filifactor alocis*, *Gemella morbillorum*, *Streptococcus constellatus and Streptococcus intermedius* have been found relevant to progressing periodontitis [13]. These differentially represented species are directly associated with the etiology of periodontitis and considered markers of periodontitis. Based on the polymicrobial synergy and dysbiosis model (PSD), periodontitis is not caused by a specific microbial species, but rather inflammation and host-microbe interactions change upon colonization with keystone pathogens [13]. More importantly, the etiology of periodontitis is further influenced by the pathogenic factors that drive microbial homeostasis versus homeostatic imbalances in oral microbial communities. In this study, we focused on the contribution of the most well-known and well characterized periodontal pathogens on oral cancer and their potential regulatory mechanisms involved in oral tumorigenesis.

Oral spirochetes, including *T*. *denticola*, often predominate in periodontal disease, though they are typically below detectable levels in healthy gingival plaque [14, 15]. The levels of *T*. *denticola* increase with the severity of periodontitis, underscoring its major role in the disease [16, 17]. *T*. *denticola* virulence stems, in part, from its protease complex dentilisin. Dentilisin contributes to *T*. *denticola* adherence to and cytotoxic effects on epithelial cells and fibroblasts, penetration of epithelial tissue, and it may play a role in *T*. *denticola* evasion of complement-mediated bactericidal activity [18–21]. *P*. *gingivalis* is frequently detected in patients with periodontitis and it plays a major role in the pathophysiology of the disease. *P*. *gingivalis* colonizes the subgingival crevice, where it mediates its invasive and replicative potential within various cell types including oral cancer cells [22, 23]. *F*. *nucleatum* is another important periodontal pathogen, since it is one of the most abundant oral bacteria present in biofilms from periodontal disease [24]. *F*. *nucleatum* has strong co-aggregation properties with most oral biofilm colonizers (early, middle, late), and it can help other bacteria cross the host epithelial and endothelial barrier [25–27]. *F*. *nucleatum’s* unique properties could play a key role in modulating microbial diversity and microbe-host interactions in the tumor biofilm environment.

The innate human microbiota has an intimate relationship with the human host in health and disease [28]. Multiple studies have reported that microbial dysbiosis is linked to a variety of diseases, including cancer [29, 30]. For example, colorectal cancer has been associated with a dysbiosis of host-microbial interactions [31–33]. The genus *Fusobacterium* spp. may be one of the etiological factors associated with colorectal carcinoma (CRC) [31, 32, 34–36]. Furthermore, genomic analysis of the microbiome of colorectal cancer patients revealed a significant enrichment in *Fusobacterium* species and depletion in species from the phyla *Bacteroidetes* and *Firmicutes* relative to the healthy colon tissues [31, 37]. Further, patients with colorectal cancer have identical strains of *F*. *nucleatum* in their colorectal cancer and oral cavity [38]. Investigators have also recently examined the relationship between the oral microbiota and oral cancer [39, 40]. Schmidt and colleagues reported changes in abundance of oral microbiota associated with oral cancer [41]. Recently, Shin and colleagues further demonstrated that specific microbial shifts are associated with transitions from health to disease (primary and metastatic HNSCC) [42]. Importantly, these shifts point to a reduction in *Streptococcus* species and an increase in *Fusobacterium* species in the transition from health to carcinogenesis. Similar to the gut, the human oral cavity is a reservoir for hundreds of microbial species, which together coexist as a biofilm consortium [43]. Changes in these biofilms are strongly associated with oral diseases, such as dental caries and periodontal disease[44]. Epidemiological data suggest that periodontal disease and certain periodontal pathogens could play a significant role in the etiology of cancers [39, 45, 46]. Furthermore, recent studies have revealed a significant association between periodontitis and oral cancer [39, 41, 47–50]. However, knowledge about direct links between periodontal pathogens and oral cancer and the mechanisms involved are limited.

A few reports have suggested that antimicrobial peptides or bacteriocins have cytotoxic effects against cancer cells [51–55]. Nisin is a 34-amino acid polycyclic antimicrobial peptide that is produced by Gram-positive *Lactococcus* and *Streptococcus* species [56]. Many such antimicrobial peptides are effective against closely related bacterial species; however, nisin has broad-spectrum effects, as it also inhibits the growth of Gram-negative bacteria [57, 58]. Nisin is recognized as safe for animal and human consumption and is widely used as a food preservative. Nisin is effective against oral biofilms and oral pathogenic bacteria, including those associated with periodontal disease [58]. Investigators from multiple fields have shown promising results for the use of nisin to treat bacterial infections, such as mastitis in humans and cows, *Staphylococcus aureus* infections in atopic dermatitis, respiratory tract infections, and experimental gingivitis in dogs [59–65]. Recent studies show that a nisin-biogel seems to be an appropriate approach for control of periodontal disease in dogs [66, 67]. Furthermore, we recently published a review article focused on the potential therapeutic use of nisin-producing probiotics and nisin in oral and systemic diseases [68]. Given this interesting premise, we explored the cytotoxic and antitumor properties of the antimicrobial peptide, nisin, and found that it blocks OSCC tumorigenesis [69]. We further demonstrated the translational potential of a highly pure form of nisin (nisin ZP, 95% purity), which reduced oral tumorigenesis *in vivo*, and long-term treatment with nisin ZP extended the lifespan of tumor-bearing mice [58, 70]. In addition, high purity forms of nisin ZP synergize with cisplatin to induce apoptosis in HNSCC cells that are highly resistant to ionizing radiation and cisplatin [71]. Thus, bacteriocins like nisin have both antibacterial and anticancer effects; properties that may be useful in treating cancers modified by bacterial dysbiosis.

The goal of the present study was to test the hypothesis that key periodontal pathogens promote oral cancer tumorigenesis, and that the bacteriocin nisin can mitigate these effects. This study demonstrates that key periodontal pathogens, *T*. *denticola*, *P*. *gingivalis* and *F*. *nucleatum*, enhance OSCC cell migration, invasion, stemness, and tumor aggressivity via crosstalk between integrin/FAK and TLR/MyDD88 signaling pathways, which can be abrogated by bacteriocin/nisin treatment. This study offers the first direct evidence that a bacteriocin, nisin, inhibits periodontal pathogen-enhanced oral carcinogenesis.

## Results

### Periodontal pathogens promote OSCC cell migration and invasion, and nisin ZP inhibits these processes

**Fig 1:**
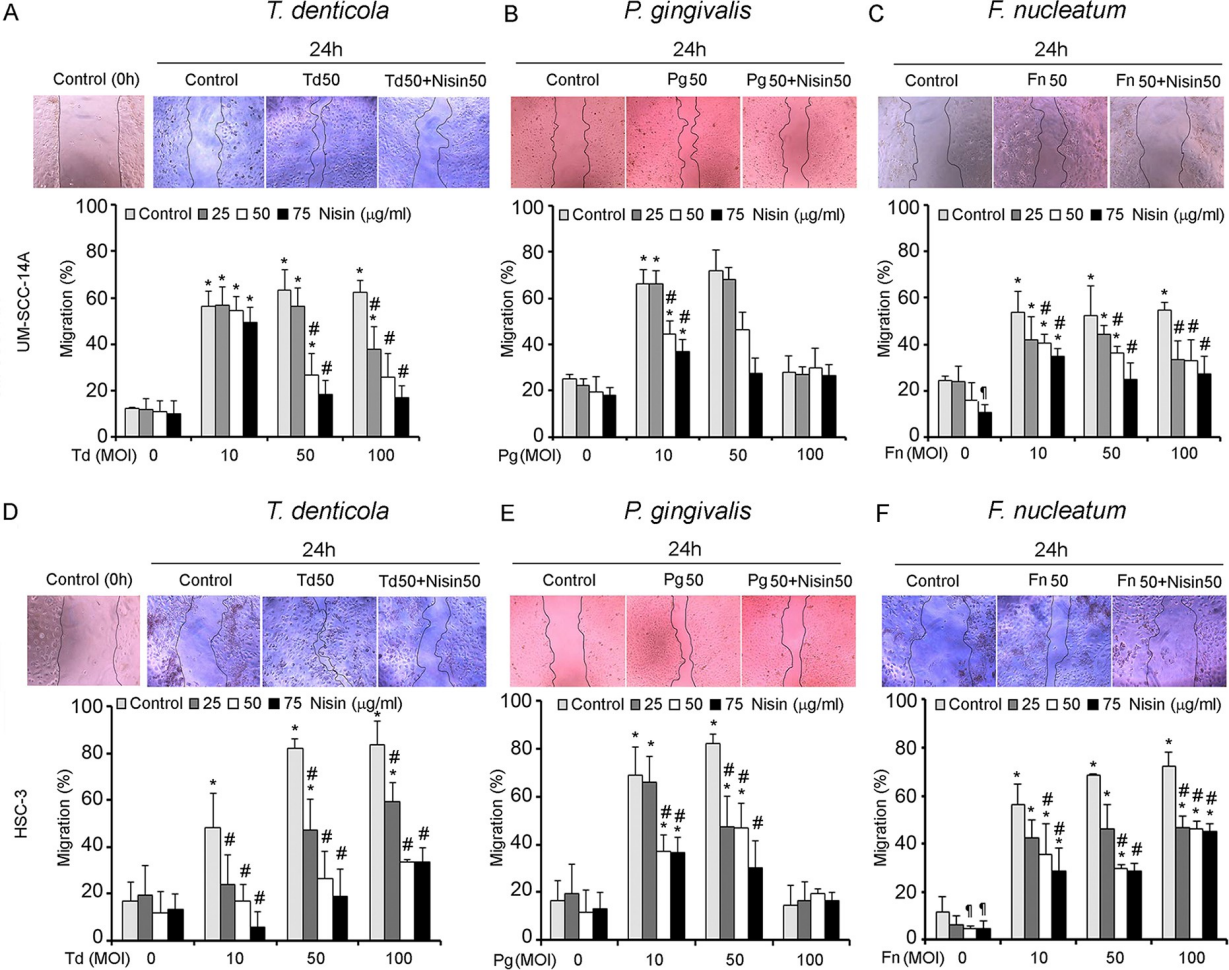
*T*. *denticola*, *P*. *gingivalis and F*. *nucleatum* promote migration of OSCC cells and nisin inhibits that process. Cells (UM-SCC-14A and HSC-3) were challenged with control medium or media containing different MOIs (10, 50 or 100) of *T*. *denticola*, *P*. *gingivalis* or *F*. *nucleatum* for 2 h then treated with nisin as indicated for 24 h. (A-F) Representative images and graphs show the total migratory distance of cells from the edges of the wounds (magnification 100x). Measurements were made after 24 h. Data represent mean ± SD from three independent experiments. *Comparison between groups relative to their media controls *p≤0.05; ^#^Comparison between groups relative to their matching concentrations of pathogens alone ^*#*^*p*≤0.05. ^¶^Comparison between groups relative to their media control *p*≤0.05.

**Fig 2:**
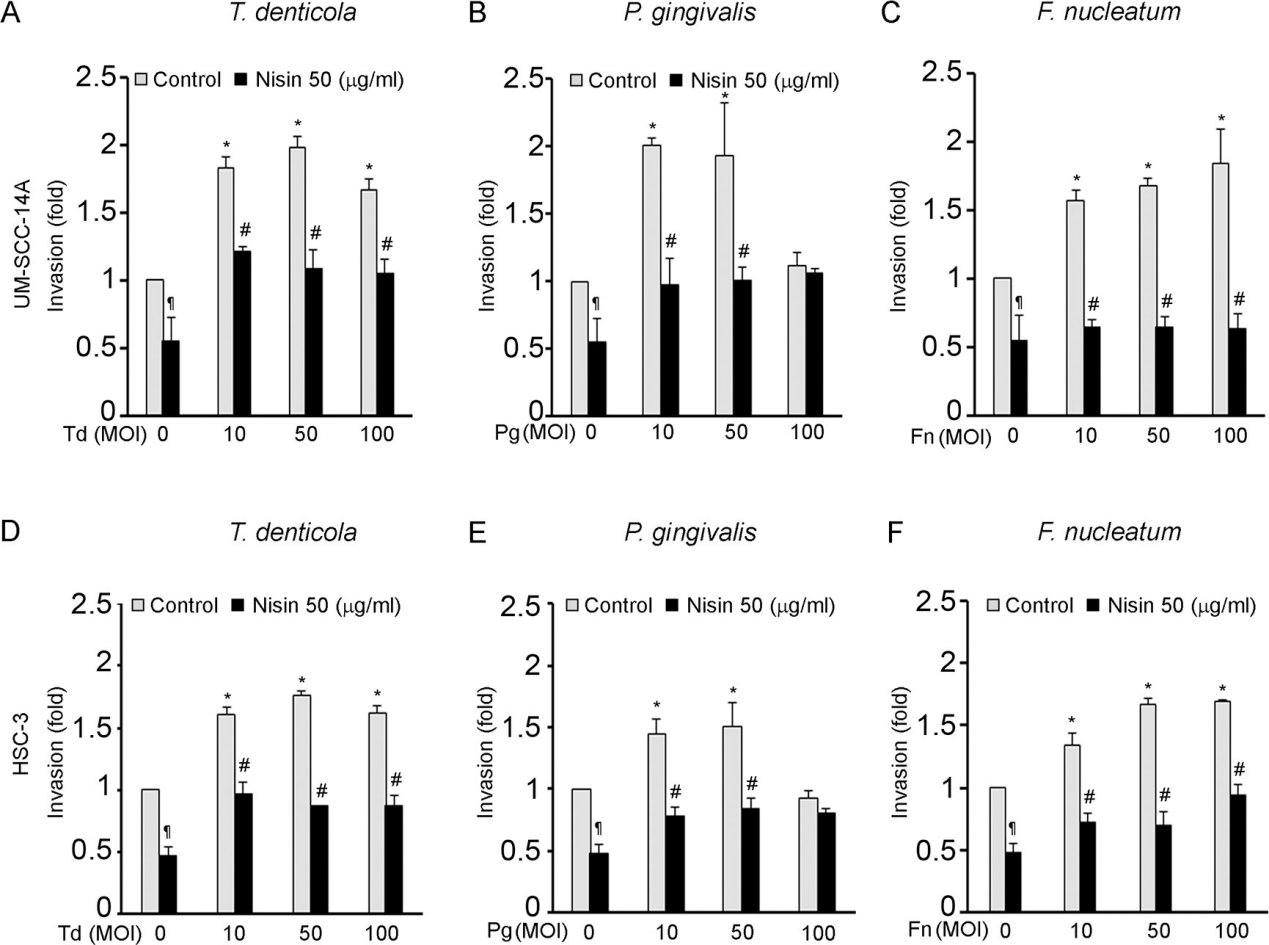
*T*. *denticola*, *P*. *gingivalis and F*. *nucleatum* promote invasion of OSCC cells and nisin inhibits that process. Cells (UM-SCC-14A and HSC-3) were challenged with control medium or media containing different MOIs of *T*. *denticola*, *P*. *gingivalis* or *F*. *nucleatum* for 2 h then treated with nisin (50 μg/ml) for 24 hours and evaluated for invasion potential. Cells that invaded the matrigel-coated membranes were labeled with AM-fluorescent dye and fluorescence intensity was read at 485/520. (A-F) Fold change in invasion relative to the unchallenged cells is illustrated in the graphs. Data represent mean ± SD from three independent experiments. *Comparison between groups relative to their media controls *p≤0.05; ^#^Comparison between groups relative to their matching concentrations of pathogens alone ^*#*^*p*≤0.05. ^¶^Comparison between groups relative to their media control *p*≤0.05.

**Fig 3:**
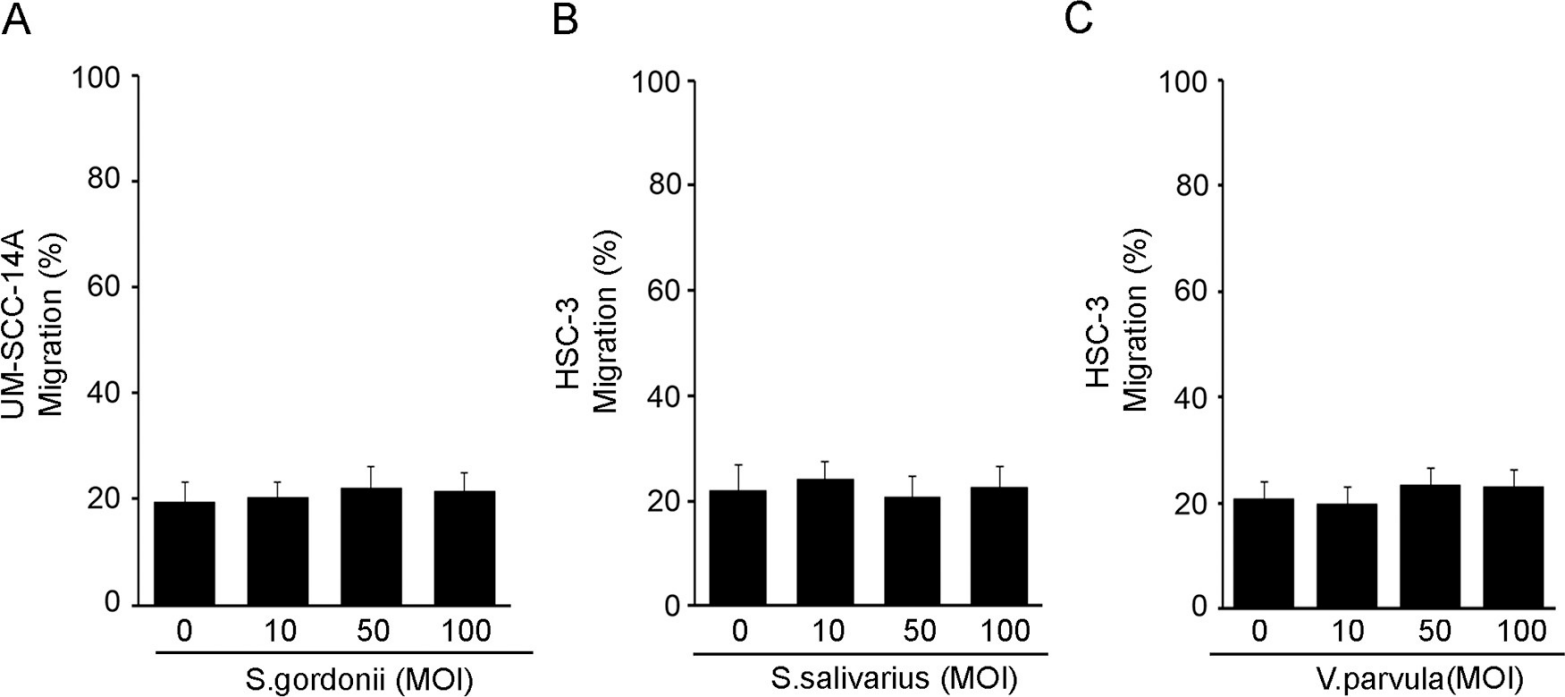
Treatment with oral commensal bacteria (*S*. *gordonii*, *S*. *salivaris* or *V*. *parvula*) does not impact OSCC cell migration. OSCC cells (UM-SCC-14A and HSC-3) were challenged with control medium or media containing different MOIs of (A) *S*. *gordonii*, (B) *S*. *salivaris* or (C) *V*. *parvula* for 2 h then cultured for 24 hours and evaluated for migratory potential. Graphs show the total migratory distance of cells from the edges of the wounds. Data represent mean ± SD from three independent experiments.

The effects of pathogenic oral bacteria, *T*. *denticola*, *P*. *gingivalis*, and *F*. *nucleatum*, on OSCC cell migration and invasion were evaluated using a scratch migration assay and matrigel invasion assay. All three periodontal pathogens significantly increased OSCC cell migration in two different OSCC cell lines (Fig 1A–1F). Similarly, all three periodontal pathogens significantly increased OSCC cell invasion (Fig 2A–2F). Addition of nisin ZP significantly inhibited the pathogen-induced cell migration (Fig 1A–1F). The nisin effects were dose-dependent. Similarly, nisin ZP significantly inhibited the pathogen-induced invasion in OSCC cells (Fig 2A–2F). However, *P*. *gingivalis* at high concentrations (100 MOI) induced apoptotic effects on OSCC cells (S2 Fig), whereas *T*. *denticola* and *F*. *nucleatum* had minimal effects on OSCC proliferation and apoptosis (S1 and S2 Figs). In contrast to the effects by periodontal pathogens, treatment of OSCC cells with commensal oral bacteria (*S*. *gordonii*, *S*. *salivarius and V*. *parvula*) did not mediate any significant changes in cell migration (Fig 3A–3C).

### Periodontal pathogens promote tumorsphere formation of OSCC cells, and nisin ZP inhibits this process

**Fig 4:**
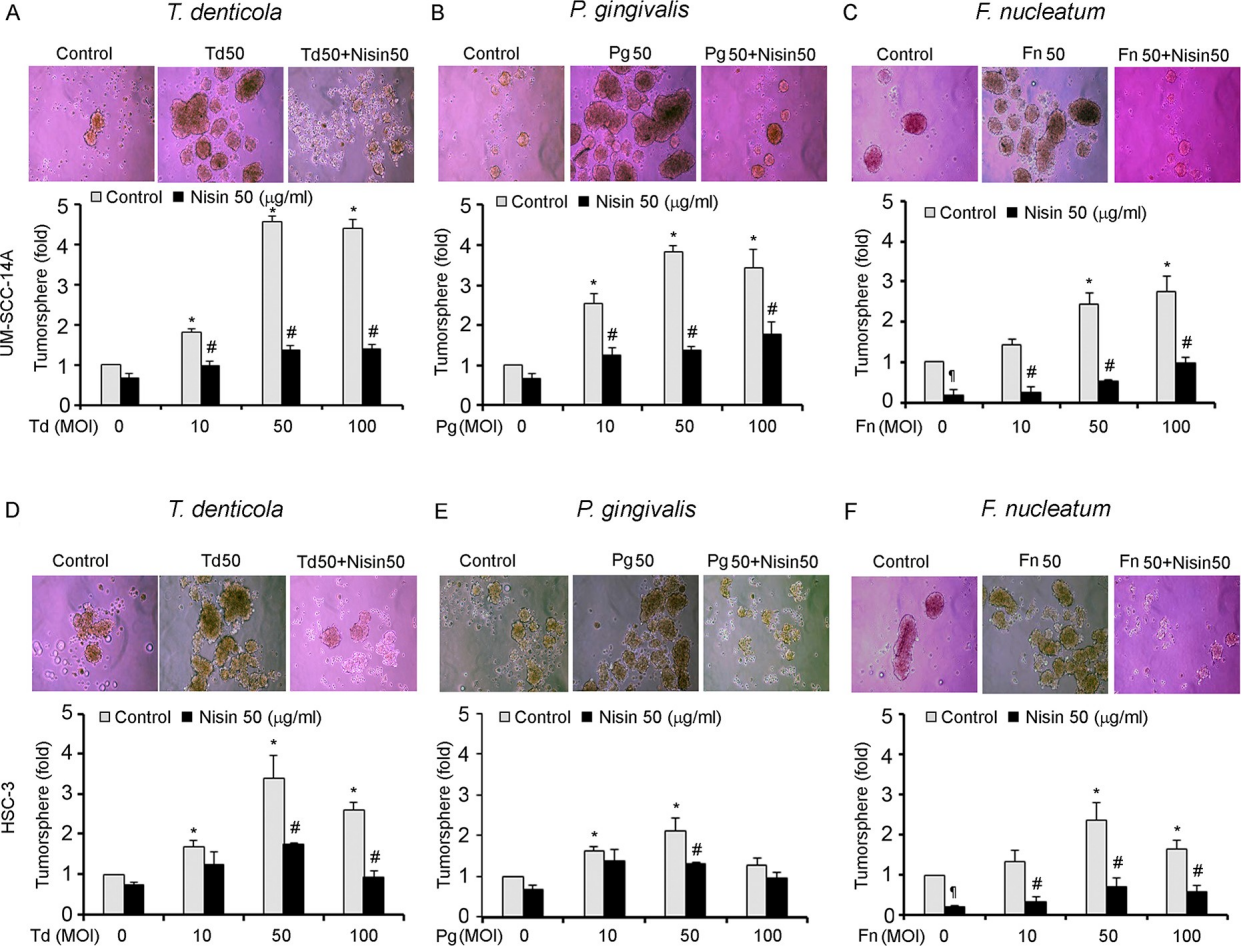
*T*. *denticola*, *P*. *gingivalis and F*. *nucleatum* promote orasphere formation of OSCC cells and nisin inhibits that process. Cells (UM-SCC-14A and HSC-3) were challenged with control medium or media containing different MOIs of *T*. *denticola*, *P*. *gingivalis* or *F*. *nucleatum* for 2 h then treated with nisin (50 μg/ml) under suspension conditions for 24 h. (A-F) Representative phase contrast images are shown, and graphs show the fold change in orasphere formation after 24 h (magnification 100x). Data represent mean ± SD from three independent experiments. *Comparison between groups relative to their media controls *p≤0.05; ^#^Comparison between groups relative to their matching concentrations of pathogens alone ^*#*^*p*≤0.05. ^¶^Comparison between groups relative to their media control *p*≤0.05.

Next, we sought to determine if periodontal pathogens might also alter the tumorsphere formation of OSCC cells. This was evaluated using tumorsphere assays on pathogen treated OSCC cells. All three periodontal pathogens significantly enhanced tumorsphere formation (Fig 4A–4F). However, *P*. *gingivalis* at high concentrations (100 MOI) appeared to be toxic to OSCC cells. We further found that treatment with nisin ZP significantly blocked pathogen-enhanced tumorsphere formation (Fig 4A–4F).

### *T*. *denticola*, *P*. *gingivalis and F*. *nucleatum* promote tumorigenesis *in vivo*

**Fig 5:**
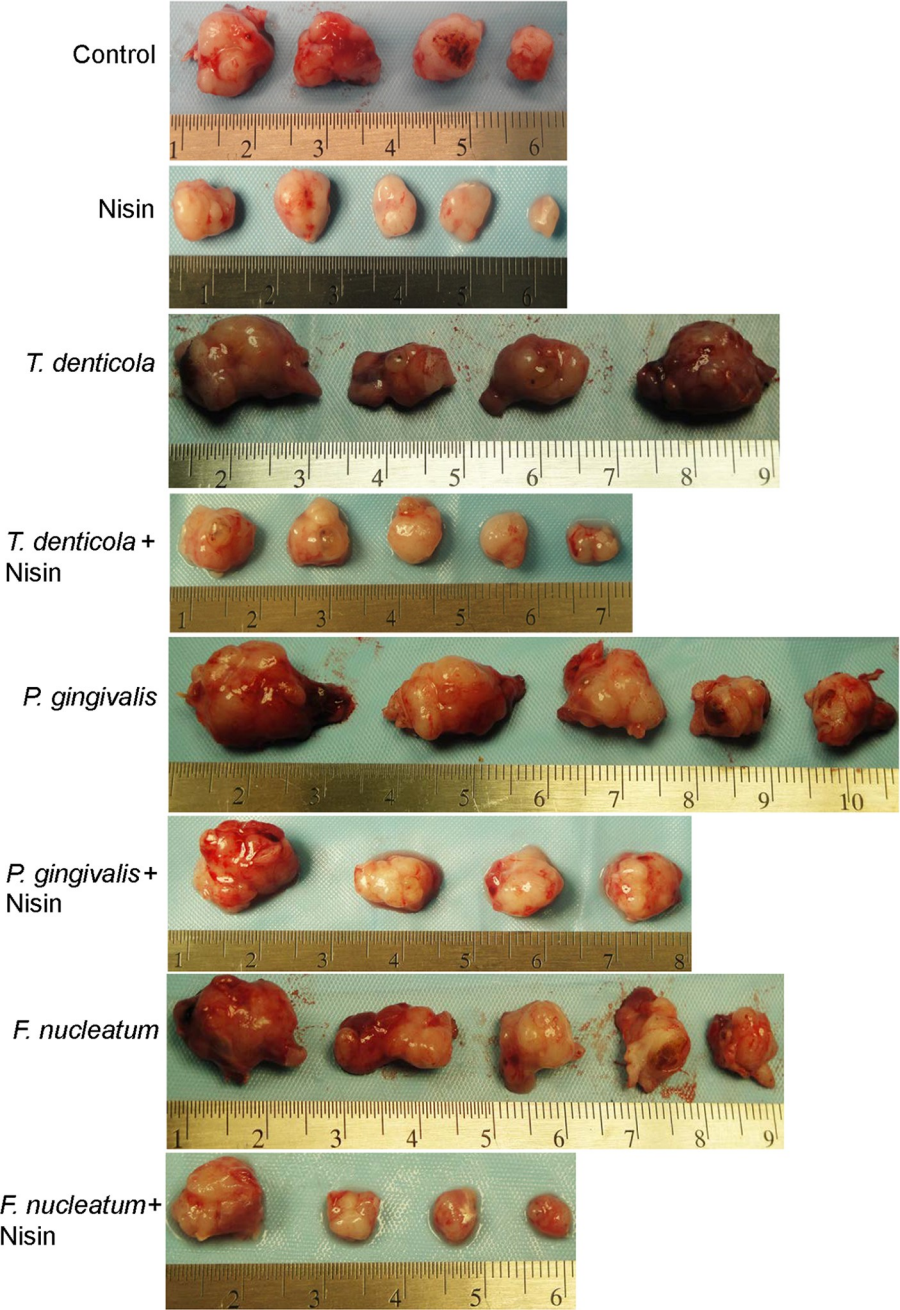
*T*. *denticola*, *P*. *gingivalis and F*. *nucleatum* promote tumorigenesis. Images show the dissected tumors obtained from mice injected with UM-SCC-14A cells challenged with control medium or media containing different MOIs of *T*. *denticola*, *P*. *gingivalis* or *F*. *nucleatum* for 2 h then treated with either water (control) or nisin (800 mg/kg body weight per day) for 7 weeks.

To investigate the *in vivo* relevance of the *in vitro* findings, a murine floor-of-mouth model that mimics human OSCC was employed [69, 70, 72, 73]. In agreement with the in vitro findings, the mouse model data showed that mice injected with pathogen-challenged OSCC cells exhibited greater tumor burden compared with the pathogen-free control counterparts (Fig 5 and Table 1). After 7 weeks of treatment, mice receiving nisin ZP (800 mg/kg body weight per day) exhibited significantly reduced tumor volumes compared to controls (Fig 5 and Table 1). Furthermore, nisin ZP significantly blocked pathogen-induced tumorigenesis (Fig 5 and Table 1). There was no significant difference in body weight between the groups (S1 Table). In addition to having no effects on mouse body weight, we reported earlier that nisin does not elicit histological signs of toxicity in the liver, lung and kidney of mice treated with 800 mg/kg body weight. Specifically, there was no histological evidence of inflammation, fibrosis, or necrosis in these organs following nisin treatment [70].

**Table 1:** Tumor volumes for mice injected with UM-SCC-14A cells challenged with control medium or media containing different periodontal pathogens (*Treponema denticola*, *Porphyromonas gingivalis* or *Fusobacterium nucleatum;* MOI: 50) then treated with water (control) or nisin (800 mg/kg body weight per day) for 7 weeks.

	Control	Nisin	*Treponema denticola*	*Treponema denticola* + Nisin	*Porphyromonas gingivalis*	*Porphyromonas gingivalis* + Nisin	*Fusobacterium nucleatum*	*Fusobacterium nucleatum* + Nisin
Animal number	Tumor volume (mm^3^)	Tumor volume (mm^3^)	Tumor volume (mm^3^)	Tumor volume (mm^3^)	Tumor volume (mm^3^)	Tumor volume (mm^3^)	Tumor volume (mm^3^)	Tumor volume (mm^3^)
1	232.7	12	582.6	219	726	265.6	441.4	267.1
2	227.8	61.3	422.6	262.1	661.5	285.7	325.4	50.6
3	147.8	29.1	477.1	128.9	541	314.2	304.2	55.6
4	99.2	118.3	419.813	105.8	193.5	176.4	151	40
5	-	19.4	-	22.6	171.5	-	191.8	-
**Mean Volume**	**176.8**	48.0*	475.5*	147.6*	458.7*	260.4*	282.7*	103.3*

Statistical analysis: Independent t-test with unequal variances

*p<0.05

### Pathogen-enhanced OSCC cell migration and tumorsphere formation are mediated via integrin alpha V/FAK signaling

**Fig 6:**
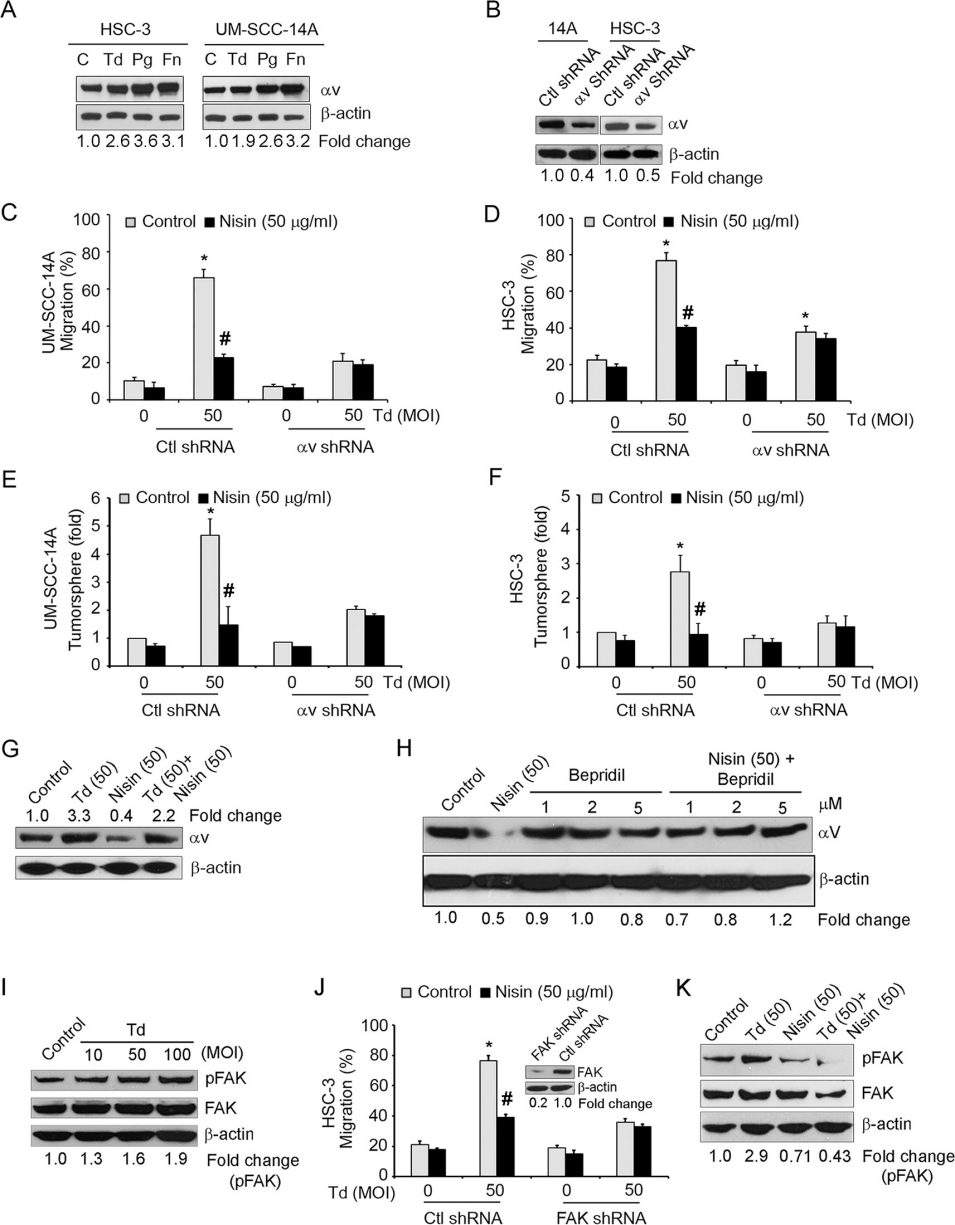
Pathogen-enhanced OSCC cell migration is mediated in part via integrin alpha V and FAK Signaling. (A) Representative immunoblots of three independent experiments showing integrin alpha V protein levels in UM-SCC-14A and HSC-3 cells challenged with control medium or media containing different MOIs of *T*. *denticola*, *P*. *gingivalis* or *F*. *nucleatum* for 2 h then cultured for 24 h. β-actin was used as a loading control for all immunoblots. (B) Representative immunoblots of three independent experiments showing integrin alpha V protein levels in cells transduced with control shRNA or alpha V shRNA. Lower panel represents the fold change values of alpha V expression using image J analysis. (C and D) Graphs show the total migratory distance of cells from the edges of the wounds. Measurements were made after 24 h. (E and F) Graphs show the fold changes in orasphere formation after 24 h. Data represent mean ± SD from three independent experiments. (G) Representative immunoblots of three independent experiments showing integrin alpha V protein levels in UM-SCC-14A cells challenged with control medium or media containing *T*. *denticola (*50 *MOI)* for 2 h then treated with nisin (50 μg/ml) for 24 h. (H) Representative immunoblots of three independent experiments showing alpha V protein levels in HSC-3 cells treated with nisin (50 μg/ml), Bepridil (1, 2, 5 μM), or nisin plus Bepridil (1, 2, 5 μM) for 24 h. (I) Representative immunoblots of three independent experiments showing phospho-FAK and FAK protein levels in HSC-3 cells challenged with control medium or media containing different MOIs of *T*. *denticola* for 2 h then cultured for 24 h. (J) Graphs show the total migratory distance of cells from the edges of the wounds. Measurements were made after 24 h. Data represent mean ± SD from three independent experiments. (Inset), Representative immunoblots of three independent experiments showing FAK protein levels in cells transduced with control shRNA or FAK shRNA. *Comparison between groups relative to their media controls *p≤0.05; ^#^Comparison between groups relative to their matching concentrations of pathogens alone ^*#*^*p*≤0.05. (K) Representative immunoblots of three independent experiments showing phospho-FAK and FAK protein levels in HSC-3 cells challenged with control medium or media containing *T*. *denticola* (50 MOI) for 2 h then treated with nisin (50 μg/ml) for 24 h.

Since integrins play critical roles in cell migration, and since integrin alpha V is central to OSCC cell migration, we investigated whether these oral pathogenic bacteria utilize host-cell integrin alpha V to trigger increased cell migration and stemness. In addition, although, it is well established that *P*. *gingivalis* and *F*. *nucleatum* are involved in periodontitis and their roles in oral cancer are emerging, little is known about the role and pathogenic mechanisms of *T*. *denticola i*n oral cancer. We therefore focused on the molecular mechanisms of *T*. *denticola* in this study. Indeed, expression of integrin alpha V was significantly upregulated upon challenge of OSCC cells with pathogenic bacteria compared to controls (Fig 6A). To further explore the potential importance of integrin alpha V in mediating pathogen-induced migration and tumorsphere formation in OSCC, we performed migration and tumorsphere assays with OSCC cells exhibiting stably suppressed levels of alpha V. As hypothesized, stable suppression of alpha V expression in OSCC cells abrogated pathogen-induced migration (Fig 6C and 6D) and stemness (Fig 6E and 6F). Since, nisin ZP rescues pathogen-enhanced OSCC cell migration and tumorsphere formation, we next examined whether nisin might be mediating these protective effects by altering the levels of integrin alpha V expression. In agreement with this proposed mechanism, nisin treatment significantly inhibited integrin alpha V expression on its own and also in the context of *T*.*denticola* challenge (Fig 6G). We next investigated the mechanism of nisin-mediated downregulation of integrin alpha V in OSCC. We examined a specific calcium channel blocker, Bepridil, in this process, since nisin is known to alter the influx of ions especially calcium influxes in HNSCC cells [69]. The downregulation of integrin alpha V triggered by nisin, is mediated, at least in part, by altered calcium influxes, since treatment with a calcium channel blocker, Bepridil, inhibits nisin-mediated downregulation of integrin alpha V (Fig 6H).

Cell migration depends on the binding of integrins to the ECM, which leads to the recruitment of focal adhesion kinase (FAK) to focal contacts and downstream signaling. In addition, integrin alpha V and FAK signaling help mediate cell migration. Therefore, we investigated the potential role of FAK in pathogen-induced cell migration. *T*. *dendicola* challenge enhanced FAK phosphorylation dose-dependently, and stable suppression of FAK abrogated pathogen-induced OSCC cell migration (Fig 6I and 6J). Furthermore, nisin ZP significantly inhibited the integrin alpha V expression and FAK phosphorylation mediated by *T*. *denticola* challenge (Fig 6G and 6K).

To further explore the underlying mechanism by which oral bacterial pathogens promote OSCC cell migration, we examined the potential role of purified bacterial factors on this process. Specifically, we performed migration assays using OSCC cells treated with purified *T*. *denticola*-lipooligosaccharide (LOS), *P*. *gingivalis*-lipopolysaccharide (LPS) and *F*. *nucleatum*-LPS. LOS and LPS derived from *T*. *denticola*, *P*. *gingivalis* and *F*. *nucleatum* promoted significant OSCC cell migration (S3A Fig). However, LPS derived from commensal oral bacteria (*V*. *Parvula*) did not show any significant changes in cell migration (S3B Fig).

### *T*. *denticola* triggers TLR/MyD88 and integrin/FAK crosstalk to mediate OSCC cell migration

**Fig 7:**
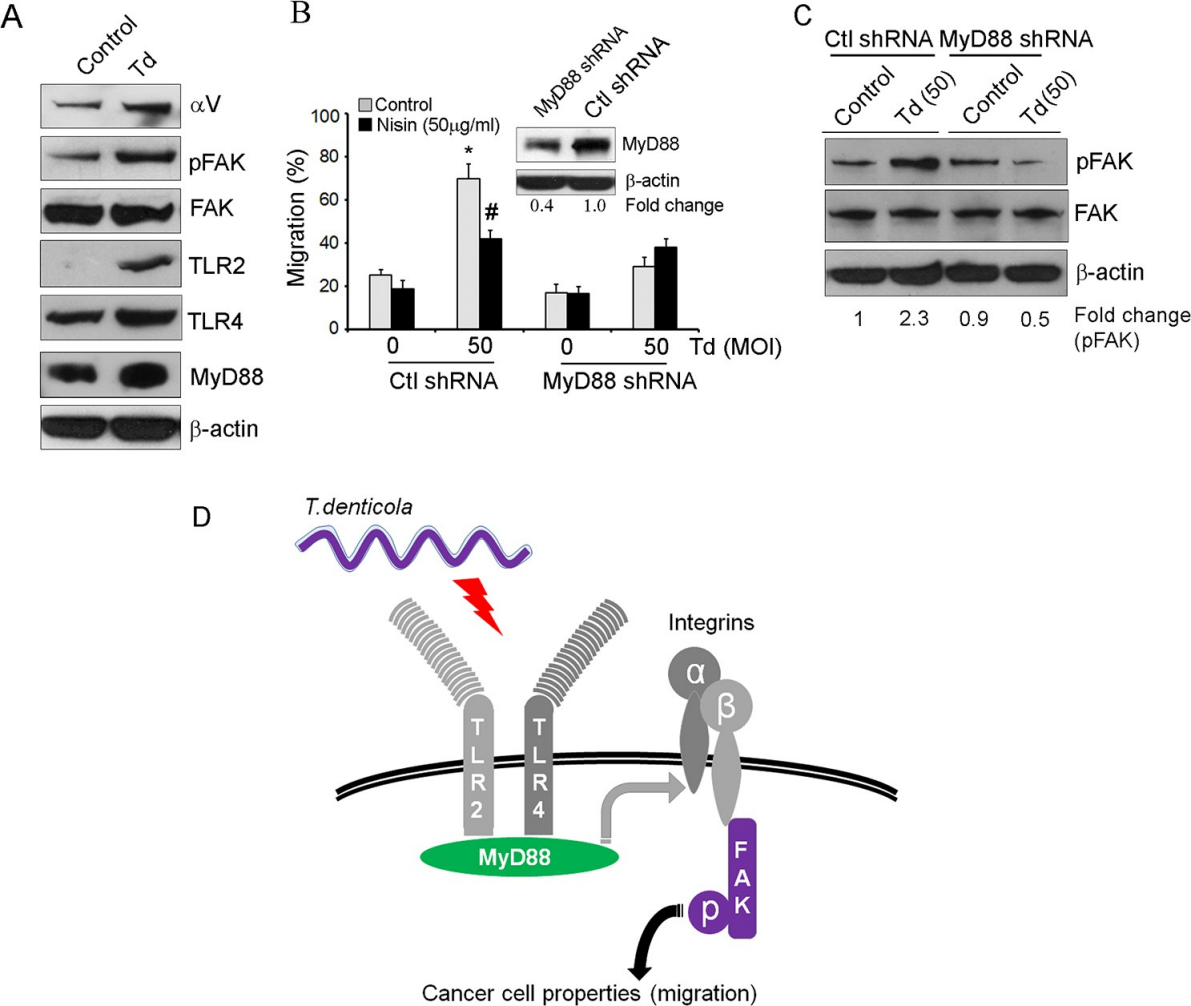
Crosstalk between TLR/MyD88 and integrin/FAK signaling contributes to the OSCC carcinogenesis phenotype. (A) Representative immunoblots of three independent experiments showing integrin alpha V, pFAK, FAK, TLR2, TLR4 and MyD88 protein levels in HSC-3 cells challenged with control medium or media containing *T*. *denticola* (50 MOI) for 2 h then cultured for 24 h. β-actin was used as a loading control. (B) Cells were challenged with control medium or media containing *T*. *denticola* (50 MOI) for 2 h then treated with nisin for 24 h and evaluated for changes in cell migration. Graphs show the total migratory distance of cells from the edges of the wounds. Measurements were made after 24 h. (Inset) Representative immunoblot of MyD88 protein levels in cells transduced with control shRNA or MyD88 shRNA. *Comparison between groups relative to their media controls *p≤0.05; ^#^Comparison between groups relative to their matching concentrations of pathogens alone ^*#*^*p*≤0.05. (C) Representative immunoblots of three independent experiments showing pFAK and FAK protein levels in cells challenged with control medium or media containing *T*. *denticola* (50 MOI) for 2 h then cultured for 24 h. (D) Model showing the crosstalk between TLR/MyD88 and integrin/FAK signaling pathways in pathogen-driven OSCC carcinogenesis. Periodontal pathogens (*T*. *denticola*) promote migration of OSCC cells via crosstalk between TLR/MyD88 and integrin/FAK signaling pathways, and thereby contribute to a more aggressive oral cancer phenotype.

Toll-like receptors (TLRs) are pattern recognition receptors (PRRs), which play a pivotal role in the development of periodontal disease and cancer pathogenesis [74, 75]. Therefore, we examined the role of TLRs and the adaptor molecule MyD88 in *T*. *denticola*-mediated OSCC cell migration. As shown in Fig 7A and 7B, *T*. *denticola* induces increased expression of integrin alpha V, phosphorylated FAK, TLR2 and 4 and MyD88, and stable suppression of MyD88 abrogates *T*. *denticola*-induced migration. Further, since MyD88 and integrin signaling pathways are known to intersect, we investigated whether TLR/MyD88 and integrin/FAK signaling pathways engage in crosstalk upon *T*. *denticola* challenge of OSCC cells. We found that stable suppression of MyD88 prevents *T*. *denticola*-induced FAK phosphorylation, supporting the hypothesis that TLR/MyD88 and integrin/FAK crosstalk contributes to the aggressivity of the pathogen-enhanced OSCC phenotype (Fig 7C).

## Discussion

The human host and the human microbiota have co-evolved to maintain an intricate balanced relationship [76]. This relationship is often mutualistic, but physiological changes caused by aging, diet, or disease processes can alter the host-microbial interactions that cause adverse effects [28, 77]. Epidemiological data supports an association between alterations in microbial communities and numerous diseases processes, including cancer. Several lines of evidence have emerged implicating bacteria in the etiology of some cancers, such as *H*. *pylori* in gastric cancer, *C*. *trachomatis* in cervical cancer, *S*. *typhi* in gall bladder cancer, both *B*. *fragilis* and *F*. *nucleatum* in colon cancer, and *P*. *gingivalis* in pancreatic cancer [31, 33, 78–83]. Bacterial pathogens in the oral cavity responsible for chronic inflammation and chronic immune cell infiltration are an accompanying histological feature of OSCC progression [39, 84–87]. Periodontitis is also associated with an increased risk for OSCC [88, 89]. The leading bacteria at sites with periodontal destruction include members of the red complex, namely *P*. *gingivalis*, *T*. *forsythia* and *T*. *denticola* [8]. Also, *F*. *nucleatum* is one of the most abundant oral bacteria present in periodontal disease biofilms. *F*. *nucleatum* has strong intermicrobial binding properties with most oral biofilm colonizers, and it can help other bacteria cross host-epithelial and -endothelial barriers [24–27]. *P*.*gingivalis* and *F*. *nucleatum* have been implicated in the pathogenesis of several chronic diseases and cancer. Although relationships between these bacteria and the etiology and progression of colon and pancreatic cancers has been reported, direct mechanistic evidence for involvement of these oral pathogens in oral cancer is limited [31, 35, 39, 46, 81, 90–94]. The current study provides new knowledge about the involvement of *P*. *gingivalis*, *F*. *nucleatum*, and *T*. *denticola* in OSCC carcinogenesis.

Oral pathogenic bacteria contribute to oral carcinogenesis via a number of potential mechanisms. However, given the paucity of knowledge in this area, we investigated the mechanisms by which *T*. *denticola*, *P*. *gingivalis* and *F*. *nucleatum* promote oral carcinogenesis. We demonstrated that *T*. *denticola*, *P*. *gingivalis* and *F*. *nucleatum* enhanced OSCC cell migration, invasion, stemness and oral tumorigenesis, without significantly altering cell proliferation or inducing apoptosis, except *P*. *gingivalis* induced a low level of apoptosis/necrosis at a high MOI (100). These differences in response (apoptosis vs proliferation) to *P*. *gingivalis* at this high MOI likely reflect differences in the timing of these cellular processes and/or differences in the detection sensitivity of the assays. *P*. *gingivalis* can increase cell proliferation by accelerating the cell cycle via modulation of apoptosis [95]. *P*. *gingivalis* can also induce G1 cell cycle arrest and increase autophagy without affecting apoptosis [95]. Furthermore, *P*. *gingivalis* employs a selective form of autophagy mediated via an endoplasmic reticulum network in order to replicate and survive within human primary epithelial cells [96]. Thus, an autophagy mechanism mediated by *P*. *gingivalis* in infected cells may have contributed to the apoptosis. We also found that *P*. *gingivalis* at a high MOI (100) is partially toxic to HSC-3 but not to SCC-14A cells, in terms of tumorsphere formation. This likely reflects normal biological and cellular variability. In this regard, bacterial pathogens express several molecules that promote their virulence or adhesion to host cells, and these actions rely on interactions with host cell surface receptors or proteins. Differential expression of host cell receptors or proteins by HSC-3 and SCC-14A may have resulted in different cellular responses to the important *P*. *gingivalis* virulence factors, including LPS, fimbriae, gingipains, and outer membrane vesicles. Although *P*. *gingivalis* and *F*. *nucleatum* can promote migration and invasion of OSCC cells by triggering an epithelial to mesenchymal transition and acquisition of stemness properties, specific bacterial molecules or their structural features have not been specifically linked with migration effects [22, 40, 97, 98]. LPS and LOS are known to play important roles in the pathogenesis of certain bacteria, including modulation of the host cell immune response and MMP expression, however, knowledge about their role in modulating host cell/cancer cell migration is limited. *P*. *gingivalis* LPS is the best studied and it has shown heterogeneity in its acylation patterns, which result in opposite effects on TLR2/4 activation [99]. Different *P*. *gingivalis* strains express at least two different varieties of LPS mostly differing in lipid A composition (designated O-LPS and A-LPS). In addition, growth conditions influence the relative balance between LPS types in single strains [100]. LPS’s of different *F*. *nucleatum* strains show considerable diversity in LPS O-antigen composition and structure [101]. *T*. *denticola* LOS has a diacylglycerol lipid anchor and a hexose-hexosamine-hexose core region, but lacks heptose, 3-deoxy-d-manno-2-octulosonic acid, and β-hydroxy fatty acids, which are core components in LPS [102, 103]. Thus, there are some similarities between *P*. *gingivalis* and *F*. *nucleatum* LPS and *T*. *denticola* LOS that may help promote cancer cell migration. *T*. *denticola* LOS generally has decreased effects compared to the classic *E*. *coli* LPS, and not much is known about its host cell effects [102]. *P*. *gingivalis* can also promote invasion in OSCC and esophageal cancer [23, 104]. A few studies showed that infection with *P*. *gingivalis* promotes progression in chemically induced OSCC tumorigenesis and resistance to paclitaxel [105–107]. Nieman and colleagues recently reported that the *T*. *denticola* dentilisin proteinase (Td-CTLP) contributes to oral tonsillar and esophageal squamous cell carcinoma, gastric, pancreatic and colon carcinoma [108]. Although these studies demonstrated the role of periodontal pathogens in oral cancer, insights into their in vivo significance and potential pathogenic mechanisms have not been elucidated. In the current study, a well-established orthotopic mouse model of oral cancer was used to provide experimental evidence that the presence of *T*. *denticola*, *P*. *gingivalis* and *F*. *nucleatum* in the tumor microenvironment modify the biological behavior of OSCC cells and confer an aggressive phenotype in oral carcinogenesis.

Integrins are a large family of heterodimeric transmembrane receptors that mediate cell-extracellular matrix (ECM) interactions. Integrins serve as anchoring molecules by mediating the adhesion of the cellular cytoskeleton to the ECM [109]. Cancer pathogenesis and metastasis is mediated by changes in cancer cell adhesion, migration, and invasion, which are carried out by integrins. Under pathological states, defective integrin signaling can result in abnormal cell division, migration and adhesion, which are hallmarks of cancer and metastasis [110, 111]. Integrin overexpression plays a role in metastasis in several malignancies. Additionally, integrin alpha V beta 6 is highly upregulated in carcinomas of the liver, colon, GI tract, pancreas, ovary and oral cavity/OSCC [112–117]. Integrin alpha V beta 3 has been implicated in the growth of lymphoid tumor cells and melanoma *in vivo* [118, 119]. Further, alpha V beta 6 inhibition strongly interferes with migration, invasion and proliferation of tumor cells *in vitro* and HNSCC tumor growth *in vivo* [117, 120, 121]. Ahmedah and colleagues recently reviewed the implications of integrin expression and function in promoting tumor growth, invasion metastasis and neo-angiogenesis in HNSCC [122]. In an effort to delineate the molecular mechanisms that control pathogen-mediated OSCC cell migration and stemness, we examined the potential contributions of integrin alpha V, an integrin receptor important to many OSCC cell functions. Indeed, exposure of OSCC cells to *T*. *denticola*, *P*. *gingivalis* or *F*. *nucleatum* triggered increased expression of integrin alpha V, and stable suppression of alpha V expression significantly interfered with pathogen-enhanced migration and stemness of OSCC cells *in vitro*. These findings confirmed that oral pathogenic bacteria mediate a more aggressive OSCC cellular phenotype by modulating integrin alpha V expression. However, the involvement of additional integrins or other cell surface receptors cannot be excluded.

Growth factor-mediated FAK phosphorylation promotes cellular adhesion, migration, and survival; all key factors in carcinogenesis. Furthermore, increased expression of phosphorylated FAK (phosphorylation at Tyr 576) enabled identification of HNSCC tumors with a more aggressive phenotype [123]. In addition, increased FAK mRNA levels and dysregulation of integrin-FAK signaling in HNSCC led to enhanced cancer cell migration and invasion [124]. Also, proteomic analysis identified ptk2/FAK overexpression as a biomarker of radioresistance in locally advanced HNSCC, and ptk2/FAK inhibition radiosensitized HNSCC cells [125]. In agreement with these studies, the current study findings indicate that the key periodontal pathogen, *T*. *denticola* enhances OSCC cell migration and tumorsphere formation via integrin/FAK-dependent signaling.

Toll-like receptors have been implicated in a variety of cancers, including colorectal cancer, breast cancer, gastric cancer, nasopharyngeal carcinoma and oral squamous cell carcinoma.[75, 126–130]. Clinical studies have shown a significantly higher expression of TLR4 and MyD88 in oral epithelial dysplasia and OSCC [130–132]. The activation of TLR4 promotes integrin alpha V beta 3-mediated adhesion and migration of breast cancer cells [133]. Based on our proposed hypothesis, findings, and broader literature, we draw a model as shown in Fig 7D, which depicts the potential direct and indirect association of molecules in pathogen-driven OSCC carcinogenesis. Our findings demonstrated that *T*. *denticola*-induced migration of OSCC cells is regulated via TLR/MyD88 triggered activation of integrin/FAK signaling. Taken together, *T*. *denticola* promotes OSCC carcinogenesis properties, such as migration, via crosstalk between TLR/MyD88 and integrin/FAK signaling pathways, and thereby contributes to a more aggressive oral cancer phenotype.

The role of bacteriocins has not been explored in the context of modulating pathogen-mediated carcinogenesis nor in modulation of host cell receptors, such as integrins. The aggregate of microorganisms that reside in the saliva and oral mucosa and the interbacterial interactions and diversity among the microorganism are indicative of oral health [134]. Within the oral cavity, there are commensal organisms with antagonistic and probiotic potential against periodontopathogens [135]. Oral commensal bacteria that have exerted antagonistic effects against periodontal pathogens, especially against red complex bacteria include, *Staphylococcus aureus* and *Streptococcus mutans* isolates, which inhibited the growth of *T*. *denticola* and *P*. *gingivalis* by producing a bacteriocin-like inhibitory substance and lactic acid [136]. The adhesion of *P*. *gingivalis* strains was inhibited by *S*. *sanguinis*, *S*. *cristatus*, *S*. *salivarius*, *S*. *mitis*, *Actinomyces naeslundii*, and *Haemophilus parainfluenzae [137, 138].* Also, clinical isolates from healthy people containing growth-inhibiting strains including, *Streptococcus*, *Actinomyces*, and *Bifidobacterium* strains, inhibited *P. gingivalis [135].* Nisin is a polycyclic antimicrobial peptide (bacteriocin) produced by the commensal bacterium *Lactococcus lactis* [56]. Recently, we showed that nisin reduces oral tumorigenesis, and long-term treatment with nisin extends the lifespan of tumor-bearing mice [70]. In the present study, we demonstrated that a bacteriocin, nisin, abrogates pathogen-enhanced migration, invasion, tumorsphere formation, and oral tumorigenesis *in vivo*. Furthermore, we discovered that nisin decreases integrin alpha V expression and FAK phosphorylation and the concomitant negative cancer cell phenotype triggered by *T*.*denticola*. Nisin induces lipid II aggregation, thereby causing increased membrane permeability of the targeted cell. Specifically, it exerts its inhibitory activity by inhibiting bacterial cell wall biosynthesis and mediating stable pore formation in the target membrane [139, 140]. Our previous studies have demonstrated that this mechanism of action is also present in cancer cells [69]. The mechanism by which nisin modulates integrin alpha V and FAK signaling is not known, although nisin is known to embed itself into lipid membranes to mediate pore formation and change ion fluxes. Thus it is possible that nisin is similarly altering OSCC cell membrane structure and ion fluxes that impact integrin/FAK signaling [69]. As hypothesized, in this study, we provide further evidence that nisin alters calcium ion influxes and thereby downregulates alpha V expression. Thus, nisin, inhibits periodontal pathogen (*T*. *denticola*)-mediated oral carcinogenesis by downregulating integrin alpha V expression and FAK phosphorylation. This suggests that integrin alpha V/FAK signaling could be a novel target and nisin a novel agent in the prevention of periodontal-pathogen (*T*. *denticola*)-enhanced oral tumorigenesis.

In summary, our findings demonstrate that a periodontal pathogen (*T*. *denticola*)-rich environment profoundly influences the tumor microenvironment by enhancing TLR/MyD88 and integrin/FAK crosstalk and signaling pathways. These signaling events trigger enhanced cancer cell migration and tumorsphere formation; properties that all contribute to a more aggressive OSCC phenotype. Overall, the present study provides novel evidence and insights into the molecular mechanisms of periodontal pathogen (*T*. *denticola*)-driven oral tumorigenesis and implicates the contribution of Toll-like receptors in the regulation of extracellular matrix and integrins as part of this process. Nisin alters these pathogen-mediated effects and mechanisms in OSCC, and since nisin also inhibits oral pathogenic bacteria and oral carcinogenesis directly, nisin has a broad therapeutic potential as an antimicrobial and as an anti-cancer agent. Altogether our findings shed new light on molecular mechanisms of pathogen-mediated carcinogenesis and pave the way to better understand the anticancer potential of the bacteriocin, nisin in oral carcinogenesis.

## Materials and methods

### Cancer cell culture

Two human OSCC cell lines were used for these studies. OSCC cell line authentication and origin was provided by their sources and published extensively. The human OSCC cell lines, UM-SCC-14A (floor of mouth) and HSC-3 (tongue) were provided by Dr. Thomas Carey (Professor, University of Michigan, MI) and by Dr. Randall Kramer (Professor, University of California, San Francisco, CA), respectively [141, 142]. OSCC cells were maintained in Dulbecco’s modified Eagle’s medium (DMEM) containing 10% fetal bovine serum, 1% penicillin, and 1% streptomycin at 37°C in 5% CO_2_.

### Bacterial culture

Anaerobic bacteria *T*. *denticola* (ATCC 35405), *P*. *gingivalis* (ATCC 33277) and *F*. *nucleatum* (ATCC 25586) were purchased from ATCC. *T*. *denticola*, *P*. *gingivalis* and *F*. *nucleatum* were grown as described previously at 37°C under oxygen deprived anaerobic conditions in New Oral Spirochete Medium or in Brain-heart Infusion (BHI) broth supplemented with hemin (5 μg ml^-1^) and vitamin K (1 μg ml^-1^) [143, 144]. Purity of spirochete cultures was confirmed by dark field microscopy prior to use in experiments. Oral commensal bacteria *S*. *gordonii* (ATCC 10558), *S*. *salivarius* (ATCC 13419) and *V*. *parvula* (ATCC 10790) were grown overnight at 37°C under anaerobic conditions in BHI broth then harvested for subsequent assays. Gram staining followed by microscopic evaluation and colony morphology on Mitis Salivarius plates were used to confirm purity of cultures. *T*.*denticola* lipoologosaccharide (Td-LOS) was a gift from Daniel Grenier, Universite Laval, Quebec, Canada, *P*.*gingivalis* lipopolysaccharide (Pg-LPS) and *F*.*nucleatum* lipopolysaccharide (Fn-LPS) were provided by Richard P. Darveau, University of Washington, Seattle and *V*. *parvula* lipopolysaccharide (Vp-LPS) was provided by Christopher Fenno, University of Michigan, Ann Arbor, MI.

### Nisin

High content nisin ZP (95% ultrapure; % weight/weight; hydrous potency ≥38,000 IU/mg) was purchased from Handary (Brussels, Belgium). Nisin ZP was reconstituted in water (5 mg/ml) and used for all experiments. Bepridil was obtained from Sigma-Aldrich (B5016, St. Louis, MO).

### Challenge of OSCC cells with periodontal pathogens

Bacteria in broth cultures were collected by centrifugation then re-suspended in serum- and antibiotic-free DMEM to an optical density of 0.1 at 600 nm, such that the cellular density was approximately 2.4×10^8^ cells/ml for *T*. *denticola and* 1×10^8^ cells/ml for *P*. *gingivalis and F*. *nucleatum*. Oral cancer cells (UM-SCC-14A and HSC-3) were challenged with control medium or media containing different MOIs of *T*. *denticola*, *P*. *gingivalis* or *F*. *nucleatum* for 2 h. After the two-hour challenge, cells were washed with PBS three times then treated with nisin for 24 h.

### Migration assay

Migration assays were performed as previously described [145, 146]. Cells (UM-SCC-14A and HSC-3) grown overnight were challenged with *T*. *denticola*, *P*. *gingivalis* or *F*. *nucleatum* for 2 h then washed three times with PBS. A uniform wound or scratch was then placed across the “cell lawn” using plastic pipette tips and cells were then cultured for 24 h in the presence of nisin. Standard photographs were taken of the wounded areas using NIS-Elements BR4.13.04 imaging software. The cell migration distance was calculated by subtracting the distance at the lesion edge at 24 h from the distance measured at 0 h.

### Matrigel invasion assay

To evaluate invasion, transwell inserts with 8 μm pores (Becton Dickinson) were first coated with 50 μl matrigel (BD Matrigel, BD Biosciences, San Jose, CA). Cells grown overnight were challenged with *T*. *denticola*, *P*. *gingivalis* or *F*. *nucleatum* for 2 h then seeded (1 × 10^5^) in the upper chambers of the transwells with 100 μl of serum free medium. Various concentrations of nisin ZP were placed in the lower chambers and cells were then incubated at 37°C for 24 h. Cells that invaded the matrigel-coated membrane and emerged on the underside surface of the membrane were labeled with Calcein AM fluorescent dye (BD Biosciences) as per the manufacturer's instructions. Fluorescence intensity was read at 485/520 in a fluorescence spectrophotometer (SPECTRA max M2, Molecular Devices) and reported as fold change in invasion relative to a media control.

### Tumorsphere assay

OSCC tumorspheres were prepared as previously reported [72, 73, 147, 148]. In brief, OSCC cells grown overnight were challenged with *T*. *denticola*, *P*. *gingivalis* or *F*. *nucleatum* for 2 h, then tumorspheres were developed by maintaining cells under suspension conditions on poly-HEMA coated plates (7.5 mg/ml in 95% ethanol, Sigma-Aldrich, St.Louis, MO) in the presence or absence of nisin ZP for 24 h. In this assay, cells that survive anchorage withdrawal form multicellular aggregates or tumorsphere. A tumorsphere is defined as an aggregate of cells that is at least 50 μm in diameter. The total area occupied by tumorspheres in each well was quantified for each treatment condition using NIS-Elements BR4.13.04 imaging software.

### Cell proliferation and apoptosis assays

To determine the effect of the bacterial pathogens and nisin on cell proliferation, the CyQUANT NF Cell Proliferation Assay Kit was used according to manufacturer’s instructions (Invitrogen/Life Technologies, Grand Island, NY). To determine the effect of the bacterial pathogens and nisin on apoptosis Ethidium Bromide and Acridine Orange (EB/AO) staining was used to measure apoptosis as previously described [70, 149]. EB and AO were obtained from Bio-Rad (Berkeley, CA) and Acros Organics (Geel, Belgium), respectively. Briefly, cells were challenged with *T*.*denticola*, *P*. *gingivalis* or *F*. *nucleatum* for 2 h. After a two hour challenge, cells were washed three times with PBS and then treated with or without nisin for 24 h. After treatment, cells were stained with an EB/AO reagent containing 100 μg/ml of ethidium bromide and 100 μg/ml of acridine orange in PBS. The stained cells were visualized under fluorescence microscopy and images were captured using a microscope equipped with a digital imaging system (Eclipse 50i Nikon, Melville, NY). One hundred cells were counted in each group and classified as either vital, apoptotic or necrotic based on their color and morphology.

### Western blotting

Western blot analyses were performed using an anti-alpha V antibody (ab124968, and ab112487 Abcam, Cambridge, MA), phospho-FAK antibody (ab24781, Abcam, Cambridge, MA) FAK antibody (ab131435, Abcam, Cambridge, MA), TLR2 antibody (ab213676, Abcam, Cambridge, MA), TLR4 antibody (SC-293072, Santa Cruz Biotecnology, Santa Cruz, CA), or MyD88 antibody (ab2064, Abcam, Cambridge, MA) followed by a horseradish peroxidase-conjugated anti-mouse antibody (SC-2005, Santa Cruz Biotechnology). Blots were then developed with the ECL-plus detection system (Thermo Fisher Scientific, Waltham, MA). To evaluate the samples for equal protein loading, membranes were stripped and re-probed with an anti-β-actin antibody (SC-1615, Santa Cruz Biotechnology).

### Development of OSCC cell lines exhibiting stable suppression of integrin alpha V, FAK, or MyD88

UM-SCC-14A and HSC-3 cells were transduced with alpha V-shRNA (SC-270259-V, Santa Cruz Biotechnology, Santa Cruz, CA) FAK shRNA (SC-29310-V, Santa Cruz Biotecnology, Santa Cruz, CA), MyD88 shRNA (SC-35986-V, Santa Cruz Biotechnology, Santa Cruz, CA) or scrambled-shRNA (SC-108080; Santa Cruz Biotechnology) lentiviral particles in 0.5 mL of serum-free media, then selected in 10 μg/mL puromycin (sc-108071; Santa Cruz Biotechnology, Santa Cruz, CA) for an additional 10 days. Surviving cell colonies were picked and propagated before testing for alpha V and FAK expression using Western blot analyses.

### Ethics statement

All mouse experiments were approved by the Animal Care and Use Committee at the University of Michigan and carried out according to the National Institutes of Health (NIH) guidelines for the ethical treatment of animals (UCUCA protocol #PRO00003743). These studies were performed at the University of Michigan when PK and YLK were affiliated with the University of Michigan.

### Oral cancer mouse model

To examine the *in vivo* effects of the pathogenic bacteria and nisin ZP, an oral cancer floor-of-mouth mouse model was used as previously described [69, 70, 72, 73]. Specifically, cells grown to 70% confluence were challenged with *T*. *denticola*, *P*. *gingivalis* or *F*. *nucleatum* for 2 h (50 MOI). After the 2 h challenge, cells were washed with PBS three times then suspended in DMEM, and mixed with an equal volume of growth factor-reduced Matrigel basement membrane matrix (BD Biosciences, San Jose, CA) to a final concentration of 1.0 x 10^6^/0.05 mL for injection. Six-week-old athymic nude mice (NCr-nu/nu strain, NCI, Frederick, MD) were anesthetized by intraperitoneal injection with 100 mg/kg ketamine and 10 mg/kg xylazine. A total volume of 0.05 mL of OSCC cell/Matrigel suspension was injected submucosally into the floor of the mouth. Two weeks after tumor cell injections and upon confirmation that tumors were established, animals were equally distributed into several groups: a control group that was given water (equal volume relative to treatment group) and other treatment groups that were given nisin (800 mg/kg body weight per day) by oral gavage for 7 weeks. Oral gavage is the principal and common method used in toxicology, pharmacology and drug-development studies with rodent models. The main advantage of oral gavage over other methods of oral administration is that it is the most accurate and reliable method for administering substances into the oral and gastro-intestinal tract, as it eliminates risks of variability in intake between individual animals. Also, oral delivery is less invasive, and is a more physiological and clinically relevant option for testing the efficacy of drugs for oral cancer floor-of-mouth mouse models [150, 151]. These treatments were well tolerated and mice exhibited normal weight measures throughout the study (S1 Table). Following tumor cell injections, mice were monitored on alternate days. Following completion of nisin administration, mice were euthanized by CO2 overdose and cervical dislocation, and then tumors were harvested, rinsed in phosphate-buffered saline (PBS), imaged, and fixed overnight in 10% buffered formalin.

### Statistical analysis

In general, values were expressed as means ± SD. Intergroup differences were analyzed by the analysis of variance (ANOVA) and Tukey-Kramer HSD test. All *in vitro* experiments were repeated at least three times. For the *in vivo* studies, independent t-tests with unequal variance were used. The numerical data used in all figures are included in S1 Data.

## Supporting information

S1 Table Body weights of mice. Time-course measurements of the body weights of mice injected with UM-SCC-14A cells challenged with control medium or media containing different periodontal pathogens (*Treponema denticola*, *Porphyromonas gingivalis* or *Fusobacterium nucleatum;* MOI: 50) then treated with water (control) or nisin (800 mg/kg body weight/day) for 7 weeks.
(DOCX)

S1 Fig *T*. *denticola*, *P*. *gingivalis* or *F*. *nucleatum* treatment does not impact OSCC proliferation Cells (UM-SCC-14A and HSC-3) were challenged with control medium or media containing different MOIs of *T*. *denticola*, *P*. *gingivalis* or *F*. *nucleatum* for 2 h then treated with nisin (50 μg/ml) for 24 hours and evaluated for cell proliferation. (A-F) Graphs show the fold change in proliferation. Data represent mean ± SD from three independent experiments.
(PDF)

S2 Fig *P*. *gingivalis but not T*. *denticola* or *F*. *nucleatum* induces apoptosis at high concentrations in OSCC. Cells (UM-SCC-14A and HSC-3) were challenged with control medium or media containing different MOIs of *T*. *denticola*, *P*. *gingivalis* or *F*. *nucleatum* for 2 h then treated with nisin (50 μg/ml) for 24 hours and evaluated for vital, apoptotic, and necrotic cells. (A-F) Graphs show changes in percentage of vital, apoptotic, and necrotic cells. Data represent mean ± SD from three independent experiments. *Comparison between groups relative to their media controls for apoptosis *p≤0.05.
(PDF)

S3 Fig *T*. *denticola*-LOS, *P*. *gingivalis*-LPS and *F*. *nucleatum*-LPS promote migration of OSCC cells. OSCC cells (HSC-3) were treated with control medium or media containing different concentrations of (A) *T*. *denticola*-LOS, *P*. *gingivalis*-LPS or *F*. *nucleatum*-LPS and (B) *V*. *parvula*-LPS for 2 h then evaluated for migration for 24 h. Graphs show the total migratory distance of cells from the edges of the wounds. Measurements were made after 24 h. Data represent mean ± SD from three independent experiments. *Comparison between groups relative to their media controls *p≤0.05.
(TIF)

S1 Data Excel spreadsheet containing, in separate sheets, the underlying numerical data and statistical analysis for Figure panels 1A-F, 2A-F, 3A-C, 4A-F, 6C-F, 6J and 7B.
(XLSX)
