# Supplementary material for: Periodontal pathogens promote cancer aggressivity via TLR/MyD88 triggered activation of Integrin/FAK signaling that is therapeutically reversible by a probiotic bacteriocin
Source: PLoS Pathog. 2020 Oct 1;16(10):e1008881. doi: 10.1371/journal.ppat.1008881 (PMC7529280; doi:10.1371/journal.ppat.1008881)
Supplement: S1 Table — Time-course measurements of the body weights of mice injected with UM-SCC-14A cells challenged with control medium or media containing different periodontal pathogens (Treponema denticola, Porphyromonas gingivalis or Fusobacterium nucleatum; MOI: 50) then treated with water (control) or nisin (800 mg/kg body weight/day) for 7 weeks. (DOCX) [file ppat.1008881.s001.docx]

**S1 Table. Body weights of mice.** Time-course measurements of the body weights of mice injected with UM-SCC-14A cells challenged with control medium or media containing different periodontal pathogens (*Treponema denticola, Porphyromonas gingivalis* or *Fusobacterium nucleatum;* MOI: 50) then treated with water (control) or nisin (800 mg/kg body weight/day) for 7 weeks.

| **Body weights of mice before tumor injection: range 20-23g** | | | | | | | | |
| --- | --- | --- | --- | --- | --- | --- | --- | --- |
| **1^st^ week after tumor induction** | | | | | | | | |
|  | **Control** | **Nisin** | **Treponema**  **denticola** | **Treponema**  **denticola +**  **Nisin** | **Porphyromonas**  **gingivalis** | **Porphyromonas**  **gingivalis +**  **Nisin** | **Fusobacterium**  **nucleatum** | **Fusobacterium**  **nucleatum +**  **Nisin** |
| Animal  number | Body weight  (g) | Body weight  (g) | Body  weight  (g) | Body  weight  (g)) | Body  weight  (g)) | Body  weight  (g) | Body  weight  (g) | Body  weight  (g) |
| 1 | 22 | 22 | 23 | 24 | 22 | 20 | 22 | 23 |
| 2 | 23 | 22 | 22 | 22 | 21 | 21 | 22 | 23 |
| 3 | 21 | 20 | 23 | 23 | 21 | 21 | 21 | 22 |
| 4 | 23 | 21 | 23 | 22 | 22 | 21 | 23 | 23 |
| 5 | 21 | 23 | 24 | 23 | 23 | 22 | 21 | 21 |
| 6 | 23 |  |  |  | 23 |  | 21 |  |
| **Mean**  **Weight** | **22.2** | **21.6** | **23** | **22.8** | **22** | **21** | **21.7** | **22.4** |
| **3^rd^ week after tumor induction** | | | | | | | | |
| 1 | 23 | 23 | 24 | 25 | 23 | 22 | 23 | 23 |
| 2 | 24 | 23 | 24 | 24 | 22 | 22 | 25 | 25 |
| 3 | 22 | 22 | 25 | 24 | 22 | 22 | 22 | 23 |
| 4 | 23 | 22 | 23 | 23 | 24 | 22 | 24 | 24 |
| 5 | 23 | 23 | 24 | 24 | 23 | 23 | 22 | 22 |
| 6 |  |  |  |  | 23 |  | 21 |  |
| **Mean**  **Weight** | **23** | **22.6** | **24** | **24** | **22.8** | **22.2** | **22.83** | **23.4** |
| **5^th^ week after tumor induction** | | | | | | | | |
| 1 | 24 | 23 | 25 | 25 | 23 | 22 | 23 | 23 |
| 2 | 24 | 23 | 24 | 24 | 22 | 23 | 25 | 25 |
| 3 | 22 | 22 | 25 | 25 | 23 | 22 | 22 | 23 |
| 4 | 23 | 22 | 25 | 24 | 24 | 22 | 24 | 24 |
| 5 | 24 | 23 | 24 | 24 | 23 | 23 | 22 | 22 |
| 6 |  |  |  |  |  |  |  |  |
| **Mean**  **Weight** | **23.4** | **22.6** | **24.6** | **24.4** | **23** | **22.4** | **23.2** | **23.4** |
| **7^th^ week after tumor induction** | | | | | | | | |
| 1 | 24 | 23 | 28 | 29 | 23 | 25 | 27 | 29 |
| 2 | 26 | 23 | 30 | 27 | 23 | 23 | 29 | 28 |
| 3 | 24 | 22 | 31 | 28 | 23 | 26 | 30 | 29 |
| 4 | 23 | 24 | 33 | 30 | 24 | 24 | 27 | 27 |
| 5 |  | 23 |  | 32 | 25 |  | 28 |  |
| 6 |  |  |  |  |  |  |  |  |
| **Mean**  **Weight** | **24.3** | **23** | **30.5** | **29.2** | **23.6** | **24.5** | **28.2** | **28.3** |
