# Supplementary figures and images for: Periodontal pathogens promote cancer aggressivity via TLR/MyD88 triggered activation of Integrin/FAK signaling that is therapeutically reversible by a probiotic bacteriocin
Source: PLoS Pathog. 2020 Oct 1;16(10):e1008881. doi: 10.1371/journal.ppat.1008881 (PMC7529280; doi:10.1371/journal.ppat.1008881)

A

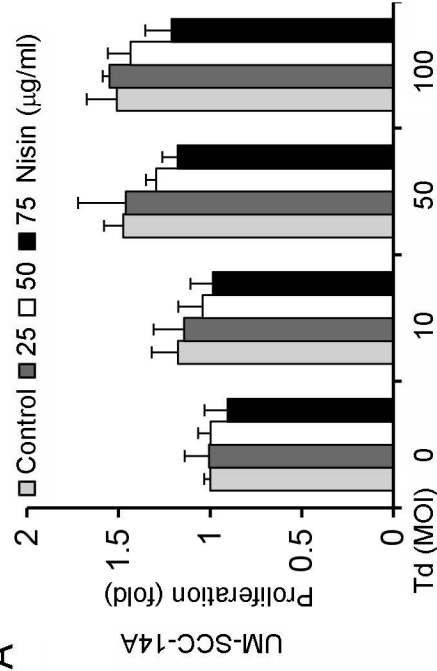

B

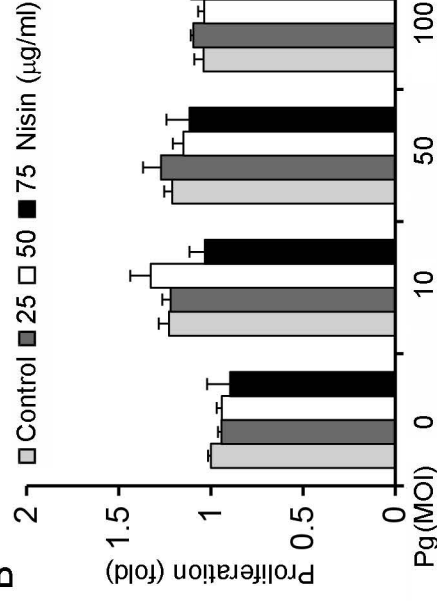

C

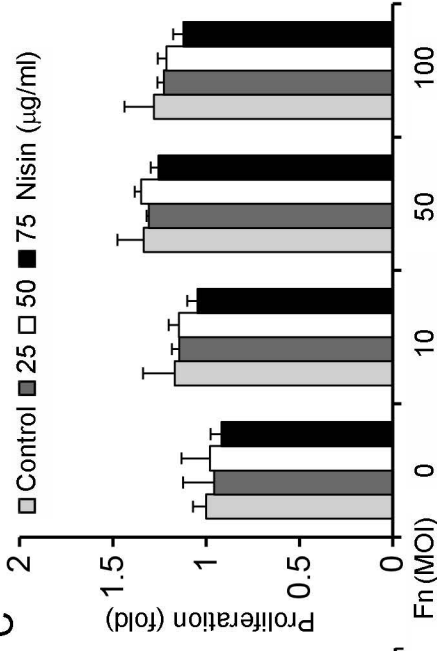

D

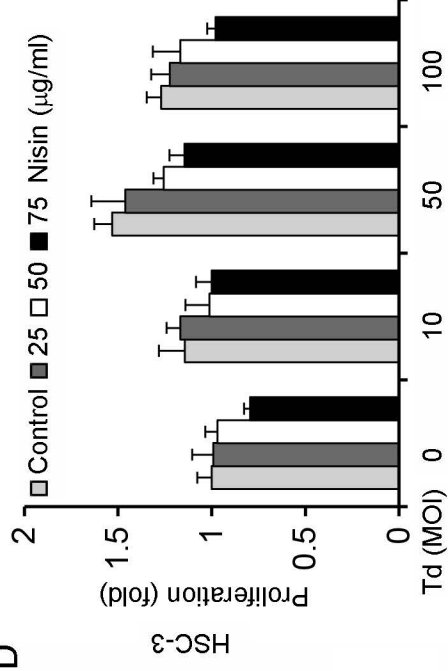

E

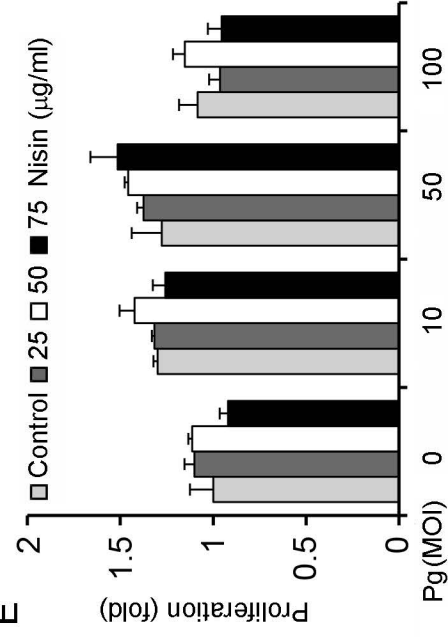

F

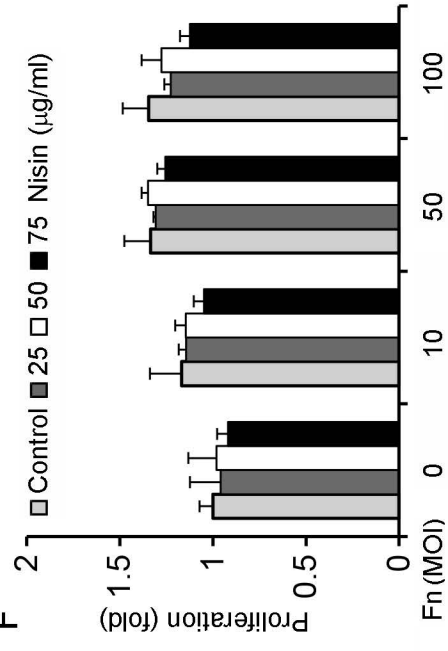

Supplement: S1 Fig — (A-F) Graphs show the fold change in proliferation. Data represent mean ± SD from three independent experiments. (PDF) [file ppat.1008881.s002.pdf]

A

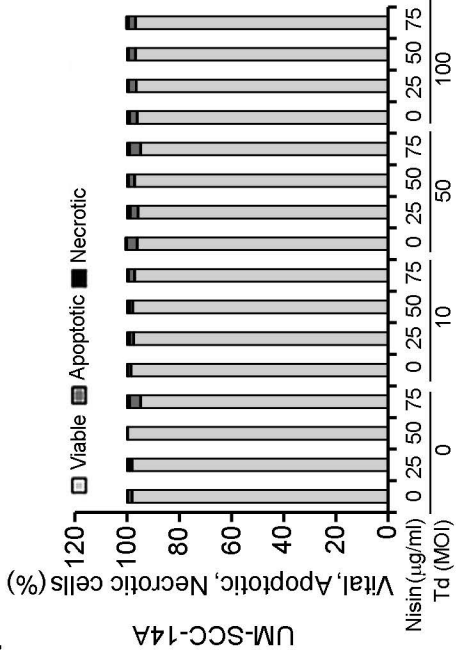

B

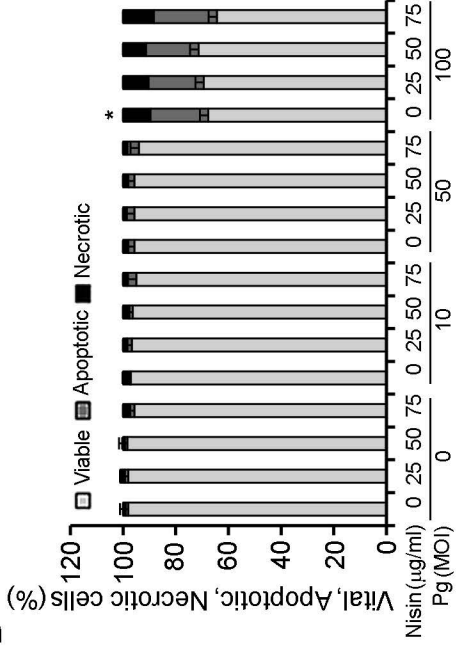

C

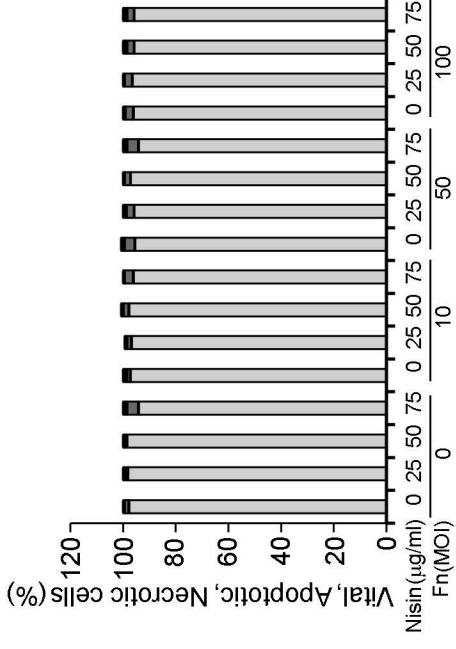

D

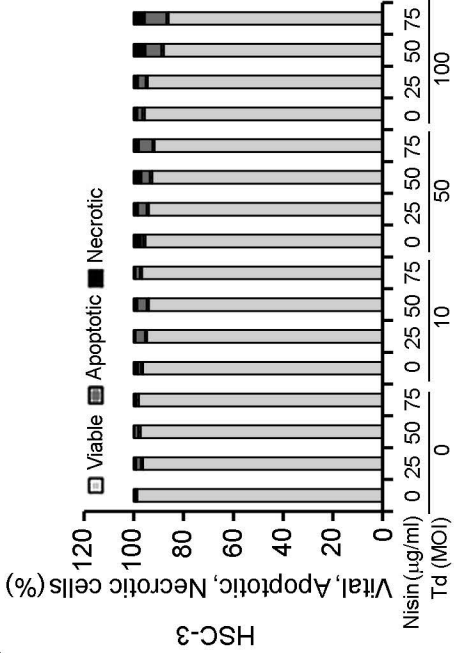

E

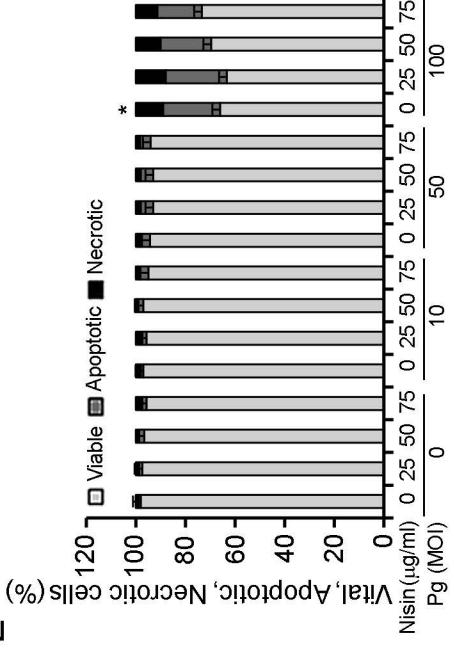

F

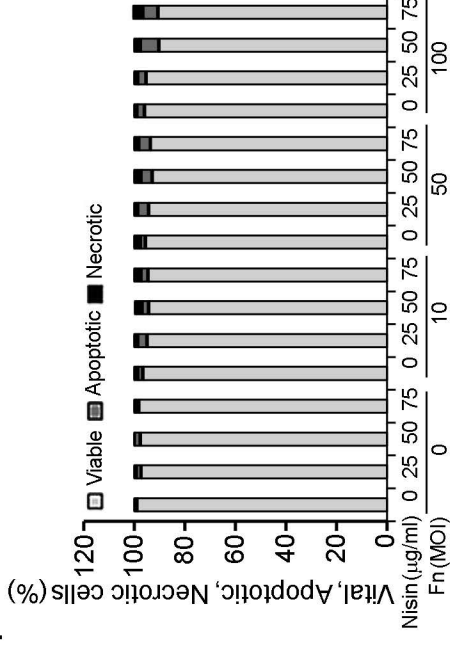

Supplement: S2 Fig — Cells (UM-SCC-14A and HSC-3) were challenged with control medium or media containing different MOIs of T. denticola, P. gingivalis or F. nucleatum for 2 h then treated with nisin (50 μg/ml) for 24 hours and evaluated for vital, apoptotic, and necrotic cells. (A-F) Graphs show changes in percentage of vital, apoptotic, and necrotic cells. Data represent mean ± SD from three independent experiments. *Comparison between groups relative to their media controls for apoptosis *p≤0.05. (PDF) [file ppat.1008881.s003.pdf]

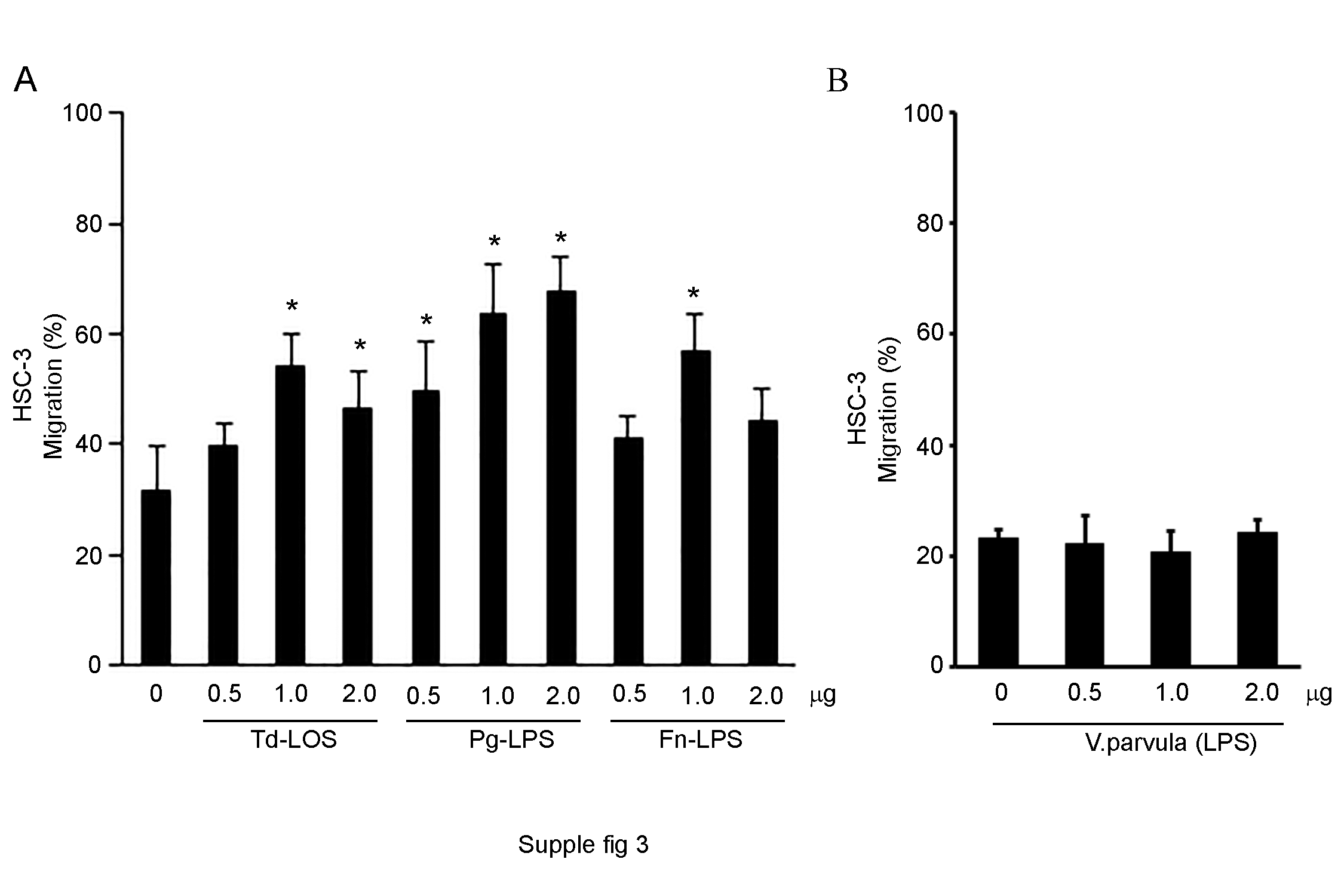

Supplement: S3 Fig — OSCC cells (HSC-3) were treated with control medium or media containing different concentrations of (A) T. denticola-LOS, P. gingivalis-LPS or F. nucleatum-LPS and (B) V. parvula-LPS for 2 h then evaluated for migration for 24 h. Graphs show the total migratory distance of cells from the edges of the wounds. Measurements were made after 24 h. Data represent mean ± SD from three independent experiments. *Comparison between groups relative to their media controls *p≤0.05. (TIF) [file ppat.1008881.s004.tif]
